# Encephalitis, High-Grade Atrioventricular Block, Acute Kidney Injury, and Hepatitis Following Atezolizumab Therapy: A Complex Case of Sequential Multiorgan Immune Checkpoint Inhibitor-Related Adverse Events

**DOI:** 10.7759/cureus.114869

**Published:** 2026-08-20

**Authors:** Umar Ismail, Bello Makama Tijjani, Susmitha D Jagadeesha

**Affiliations:** 1 Internal Medicine, Hywel Dda University Health Board, Pembrokeshire, GBR; 2 General Practice, St. Helens and Knowsley NHS Foundation Trust, Manchester, GBR

**Keywords:** 3rd degree heart block, atezolizumab, cardio-oncology, encephalitis, hepatitis, ici, immune-related adverse event (irae), nephritis, neuro oncology

## Abstract

Immune checkpoint inhibitors (ICIs) can cause serious toxicities due to widespread immune activation. We report the case of a 49-year-old man with lung cancer who presented with confusion and seizures 16 days after receiving atezolizumab. Investigations were unrevealing of a cause. Immunotherapy-related encephalitis was suspected, given the temporal association between symptoms and atezolizumab infusion. Despite treatment with antimicrobials and methylprednisolone, the patient developed ventricular standstill requiring cardiac pacing. Although neurology and cardiac rhythm improved with high-dose methylprednisolone and pacemaker insertion, the patient developed hepatitis and acute kidney injury (AKI) during steroid tapering, which resolved with re-escalation. This case highlights a potential life-threatening multiorgan dysfunction that can complicate ICI therapy. While established toxicity phenotypes present with synchronous multiorgan involvement, atypical cases may present with sequential organ involvement that can pose significant diagnostic challenges. This reinforces the need for vigilance and early multidisciplinary team coordination to ensure timely diagnosis and good outcomes.

## Introduction

Immune checkpoints are regulatory molecules that modulate the body's immune response and protect tissues from autoreactive damage [1,2]. Checkpoint molecules like cytotoxic T-lymphocyte-associated antigen 4 (CTLA4), programmed death receptor/ligand (PD1/L1), and lymphocyte activating gene 3 (LAG3) play a central role in regulating immune responses against virus-infected and transformed cells. The CTLA4 inhibits CD28-mediated T-lymphocyte co-stimulation by competing for binding to CD80/86, inhibiting T-cell activation, and suppressing interleukin-2 (IL-2) secretion and T-cell proliferation [2]. On the other hand, PD-1 is an inhibitory receptor expressed by T-cells, B-cells, natural killer (NK) cells, dendritic cells (DCs), and activated monocytes [2]. Through interactions with PD-L1 and PD-L2, PD-1 regulates effector T-cell activation and is important in maintaining peripheral immune tolerance [2]. These molecules are not only expressed by lymphocytes, macrophages, and some non-immune tissues like the heart and lungs; they are also expressed by cancer cells, which utilise them to evade immune detection.

Since their discovery, immune checkpoints have been the focus of intense research, culminating in the US FDA approval of the first immune checkpoint inhibitor (ICI) for the treatment of advanced melanoma in 2011 [2]. Immune checkpoint inhibitors are monoclonal antibodies that block CTLA4, PD-1, PD-L1 or LAG3 and induce the activation of the immune system against cancer cells. These therapies have revolutionised oncology and have improved outcomes for many patients with otherwise poor prognoses [3]. Guidelines now support their use in a variety of cancers in neoadjuvant, adjuvant and palliative settings. Despite impressive anti-tumour efficacy and a better safety profile compared to conventional systemic cancer therapies, ICIs are associated with wide-ranging toxicities termed 'immune-related adverse events (irAEs)', which can affect virtually any organ [4]. These toxicities are graded according to the Common Terminology Criteria for Adverse Events (CTCAE) into five categories based on severity, with 5 being the most severe, resulting in death [5]. 

While most irAEs are relatively benign and may in some cases predict good treatment response, some can be potentially lethal if not identified and addressed promptly [5]. Toxicities that affect the nervous system, heart, and skeletal muscles have the highest potential for devastating outcomes not only because of the vital functions performed by these organs but also due to the potential for toxicities to overlap in these tissues. The estimated incidence of neurologic, cardiac, and skeletal muscle irAEs is 1% to 5%, <5%, and 1% of patients receiving ICIs, respectively [5]. Overlapping toxicities involving the skeletal muscle (myositis), the myocardium (myocarditis), and the neuromuscular junction (myasthenia gravis) have been reported, with overall incidence below 1% [5]. While toxicities like myositis, myocarditis and myasthenia gravis (MMM) overlap syndrome are well described in the literature, toxicity phenotypes sequentially involving the central nervous system (CNS), the heart, kidneys and liver are very unusual and therefore poorly described.

Atypical patterns of toxicity need to be documented because they pose a significant diagnostic challenge for generalist physicians who are often the first to encounter them. They also represent a potential clinical blind spot that may pose significant risk to patient safety due to the high risk of misdiagnosis and misattribution. By documenting atypical cases such as this, we hope to add to the existing body of knowledge on ICI toxicities and start a wider conversation on managing diagnostic uncertainty in the age of immunotherapy as we enter a broader post-marketing surveillance period. The patient in the case described here was primarily managed at Withybush General Hospital (Haverfordwest, Wales, GBR). Pacemaker insertion was performed at our tertiary referral centre (Morriston Hospital, Swansea, Wales, GBR).

## Case presentation

A 49-year-old man with a background history of hypertension and asthma was diagnosed in March 2025 with a poorly differentiated metastatic T4N3M1c lung cancer (pleural, adrenal, and subcutaneous metastasis). Tumour immunohistochemistry confirmed PDL-1-positive membrane staining in 55% of tumour cells. Patient received palliative radiotherapy to the tumour site (pancoast tumour). Single-agent palliative immunotherapy with subcutaneous atezolizumab was planned. Cycle one of atezolizumab was given on 12th June 2025, following which the patient presented to hospital 16 days later with confusion, a flu-like illness and seizures. The seizure was initially focal, involving the face, described as twitching of the left side of the face, which later became generalised tonic-clonic, involving the whole body, with one episode lasting up to eight minutes before hospital arrival. Although there were reports from the patient's family of 'short vacant episodes' in the few months prior to the current event, there was no definite confirmation of any seizure activity or epilepsy before this admission.

On arrival to the emergency department, the patient was altered and severely agitated with a Glasgow Coma Score (GCS) of 11/15; pupils were 3 mm to 4 mm, equal and reactive bilaterally. There were no identifiable focal neurological signs. The patient was vitally unstable with elevated blood pressure, tachypnoea, severe tachycardia (heart rate 150 beats/minute) and an oxygen requirement of 2 L. Another generalised tonic seizure was witnessed by the medical team while the patient was being assessed. Seizure precautions were observed, and the patient was commenced on high-flow oxygen in the recovery position. Intravenous lorazepam 4 mg was given to abort the seizure, followed by a loading dose of levetiracetam 40 mg/kg. The patient had one episode of pyrexia (38°C) post-seizure, and a sepsis screen was completed. Blood cultures, full blood count (FBC), urea, electrolytes and creatinine (U&E), liver function tests (LFTs), troponin T, creatine kinase (CK), lactate, paracetamol/salicylate and ethanol levels were also requested, the results of which are presented in Table 1. Except for raised lactate (10.4 mmol/L), C-reactive protein (CRP) (97 mg/L) and mild troponin T elevation (26 ng/L), initial blood tests were relatively unremarkable. Initial CK and electrolytes were normal at the time of admission despite repeated tonic-clonic seizures.

**Table 1:** Lab results on admission *Although the NTproBNP specimen was sent at the time of admission, results became available three days after admission (normal turnaround time for the test in our hospital). FBC: Full blood count, WCC: White cell count, Hb: haemoglobin, MCV: Mean corpuscular volume, CRP: C-reactive protein, ALT: Alanine transaminase, CK: Creatine kinase, ANA: Anti-nuclear antibody, ANCA: Anti-neutrophil cytoplasmic antibody, RF: Rheumatoid factor, CMV: Cytomegalovirus

Test		Result	Reference range
FBC and metabolic profile	White cell count (WCC)	8.7	4-11 kU/L
	Neutrophils	6.3	1.7-7.5 kU/L
	Lymphocytes	1.5	1.1-4.8 kU/L
	Haemoglobin (Hb)	125	130-180 g/L
	Mean corpuscular volume (MCV)	85	80-100 fL
	CRP	97	<5 mg/L
	Sodium	131	133-146 mmol/L
	Potassium	5.2	3.5-5.3 mmol/L
	Urea	5.5	2.5-7.8 mmol/L
	Creatinine	84	58-110 umol/L
	Albumin	42	35-55 g/L
	Alkaline phosphatase (ALP)	273	30-130 U/L
	Alanine transaminase (ALT)	23	<50 U/L
	Bilirubin	5	<21umol/L
	Calcium	2.45	2.20-2.60 mmol/L
	Paracetamol	5	<5 mg/L
	Salicylate	Not detected	
	Ethanol	<100	
	Lactate	10.4	0.5-1.6 mmol/L
	CK	45	40-320 U/L
	Troponin T	26	<14 ng/L
	Cortisol (random)	1025	>420 nmol/L
	Phosphate	1.29	0.8-1.50 mmol/L
	Magnesium	1.01	0.70-1.00 mmol/L
	Blood culture	Negative	
	NTProBNP	2234*	<400ng/L
Autoimmune screen	Anti-nuclear antibody (ANA)	Negative	
	Anti-neutrophil cytoplasmic antibody (ANCA)	1:320 (P-ANCA)	
	Rheumatoid factor (RF)	<10	
	Anti-AMPA 1&2 and anti-GAD	Negative	
	Anti-GABAb and anti-NMDAR	Negative	
Microbiology	Borrelia IgM	Negative	
	Syphilis screen	Negative	
	Cytomegalovirus (CMV) IgM	Negative	

**Figure 1:**
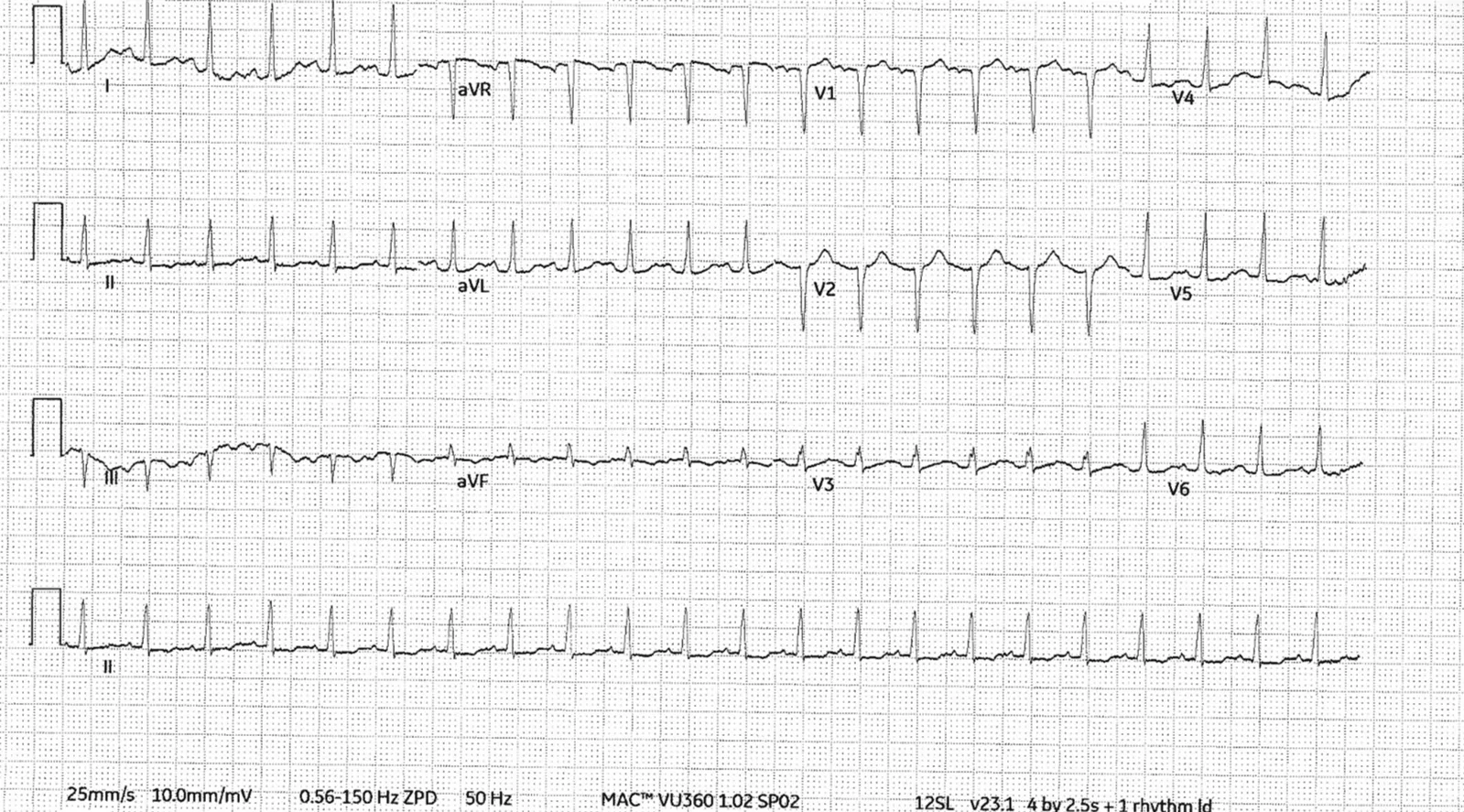
Initial ECG at time of admission showing sinus tachycardia with otherwise normal conduction ECG: Electrocardiogram

**Figure 2:**
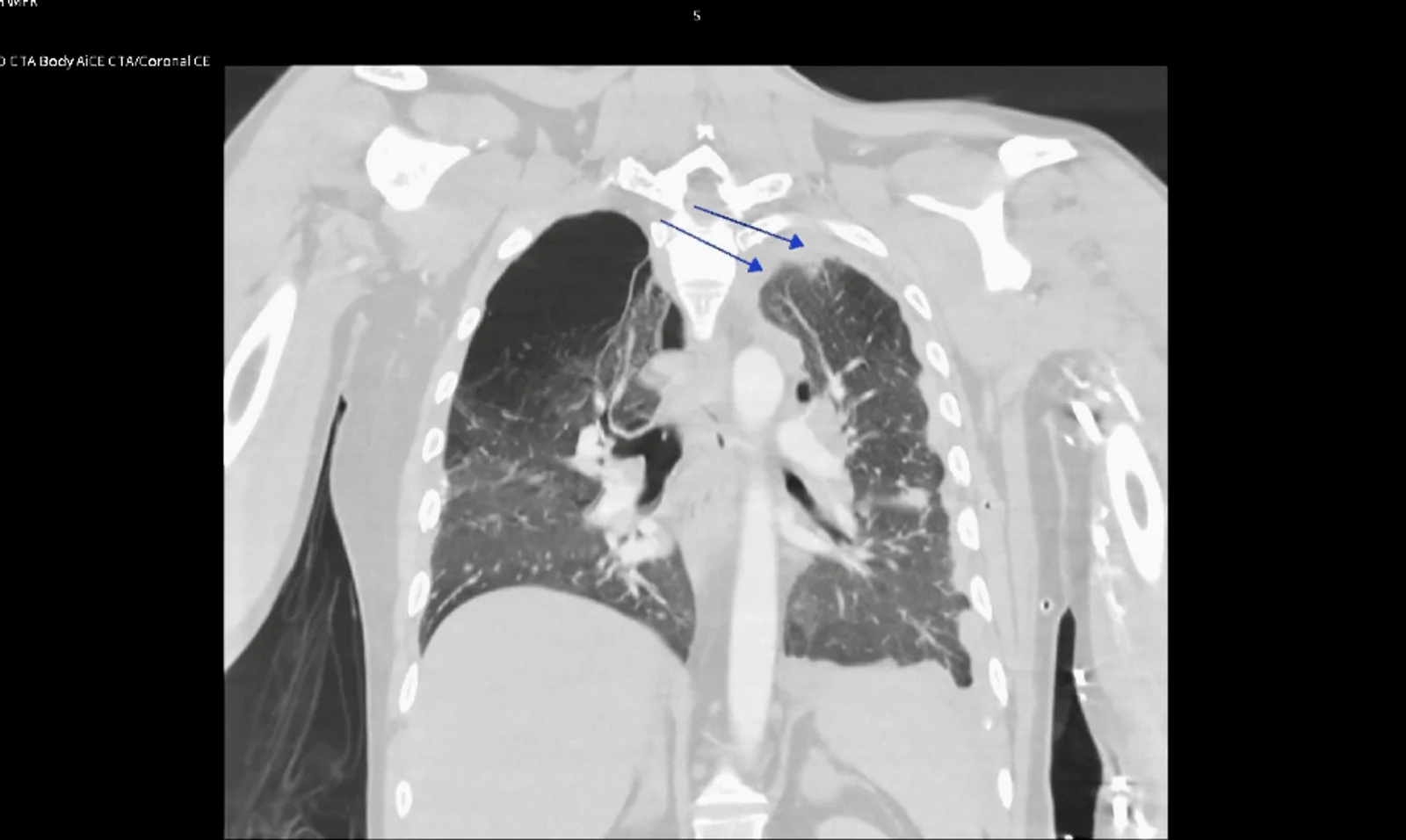
CTPA was negative for PE but showed established lung cancer (blue arrows) CTPA: Computed tomography pulmonary angiogram, PE: Pulmonary embolism

An electrocardiogram (ECG) on admission confirmed sinus tachycardia, which was assumed to be a physiologic response to hypoxia and seizures (Figure 1). Thyroid function tests (TFTs) were done and returned normal. In view of flu-like illness and confusion before seizure onset, broad-spectrum antimicrobial cover was initiated with ceftriaxone and acyclovir due to concerns about meningoencephalitis. A contrast CT of the brain and a CT pulmonary angiogram (CTPA) were requested to exclude an intracranial space-occupying lesion and pulmonary embolism (PE). The CTPA was negative for PE (Figure 2), and the CT brain was normal.

**Figure 3:**
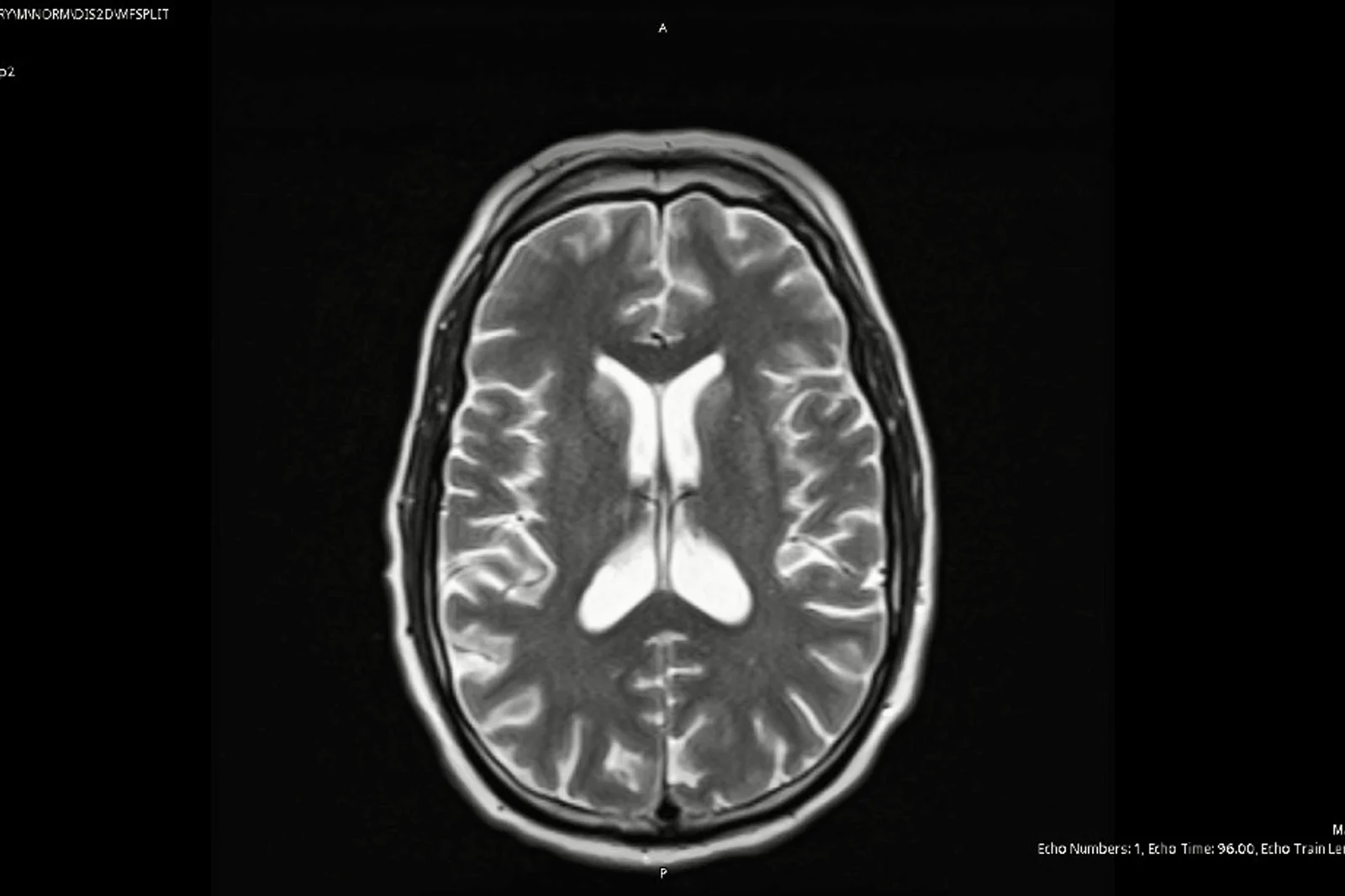
MRI of the brain is normal with no radiologic evidence of metastasis or encephalitis

Given the apparent temporal association between atezolizumab infusion and the patient's symptoms, immunotherapy-related neurotoxicity (encephalitis) was considered high on the list of differential diagnoses; this was discussed with the oncology team. Advice was given to commence IV methylprednisolone 1 mg/kg/day alongside antimicrobials and arrange an MRI of the brain, as it would help in differentiating encephalitis from leptomeningeal disease. The patient was also discussed with neurologists who felt that although immunotherapy-related encephalitis was a possibility, the list of differential diagnoses was long and advised increasing the total loading dose of levetiracetam to 4.5 g to prevent further seizures and arranged for lumbar puncture (LP) with paired protein and glucose, viral PCR, tuberculosis (TB) PCR and cultures, syphilis screen, and paraneoplastic and autoimmune encephalitis screen. Unfortunately, the patient remained severely agitated and unstable over the first 72 hours of admission, and LP could not be completed. However, an MRI of the brain was done and reported as normal (Figure 3).

**Figure 4:**
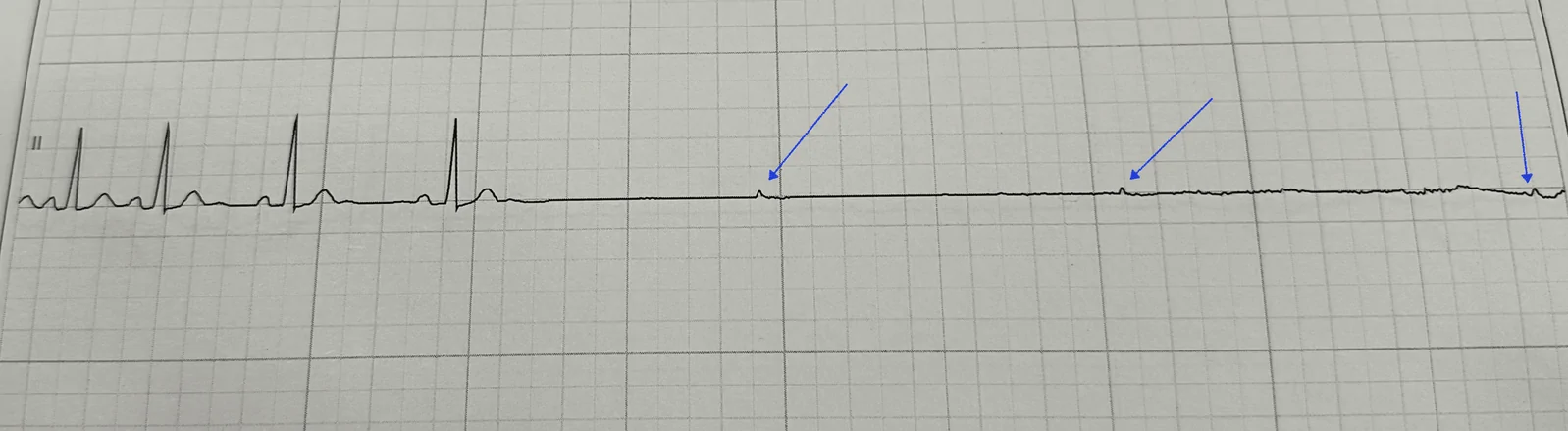
Telemetry strip showing ventricular standstill with non-conducting P-waves (blue arrows)

Further deterioration occurred by the end of day three, with the patient developing intermittent symptomatic asystole. Cardiac pauses up to 7 seconds were recorded on the cardiac monitor with evidence of ventricular standstill/non-conducting P-waves (Figure 4).

The patient was discussed with the local cardiologist, who advised commencing isoprenaline infusion and arranging transfer to a tertiary cardiac centre for pacemaker insertion. Urgent echocardiogram (ECHO) was requested to assess for other features of ICI-related cardiotoxicity. Methyprednisolone was escalated to 1000 mg/day. The ECHO was normal, but results of NTProBNP (requested at the time of admission) were markedly raised at 2234 ng/L (Table 1). The rest of day three's investigations are shown below in Table 2. Interestingly, although troponin T remained stable at 20, CK rose to 1940, which suggested striated muscle inflammation (myositis and/or myocarditis). Myocarditis was favoured given the ventricular standstill and raised NTproBNP.

**Table 2:** Results of investigations conducted on day three of hospitalisation WCC: White cell count, Hb: haemoglobin, MCV: Mean corpuscular volume, CRP: C-reactive protein, ALT: Alanine transaminase, CK: Creatine kinase

Test	Result	Reference range
WCC	6.5	4.0-11.0 kU/L
Neutrophils	5.2	1.7-7.5 kU/L
Lymphocytes	0.8	1.0-4.5 kU/L
Hb	103	130-180 g/L
MCV	78	80-100fL
CRP	28	<5 mg/L
Sodium	136	135-146 mmol/L
Potassium	3.7	3.5-5.3 mmol/L
Urea	4.7	2.5-5.7 mmol/L
Creatinine	55	umol/L
Albumin	35	g/L
ALP	172	30-130 U/L
ALT	21	<50 U/L
Bilirubin	3	<21 umol/L
Phosphate	0.79	0.8-1.50 mmol/L
Calcium	2.16	2.20-2.60 mmol/L
CK	1940	40-320 U/L

**Figure 5:**
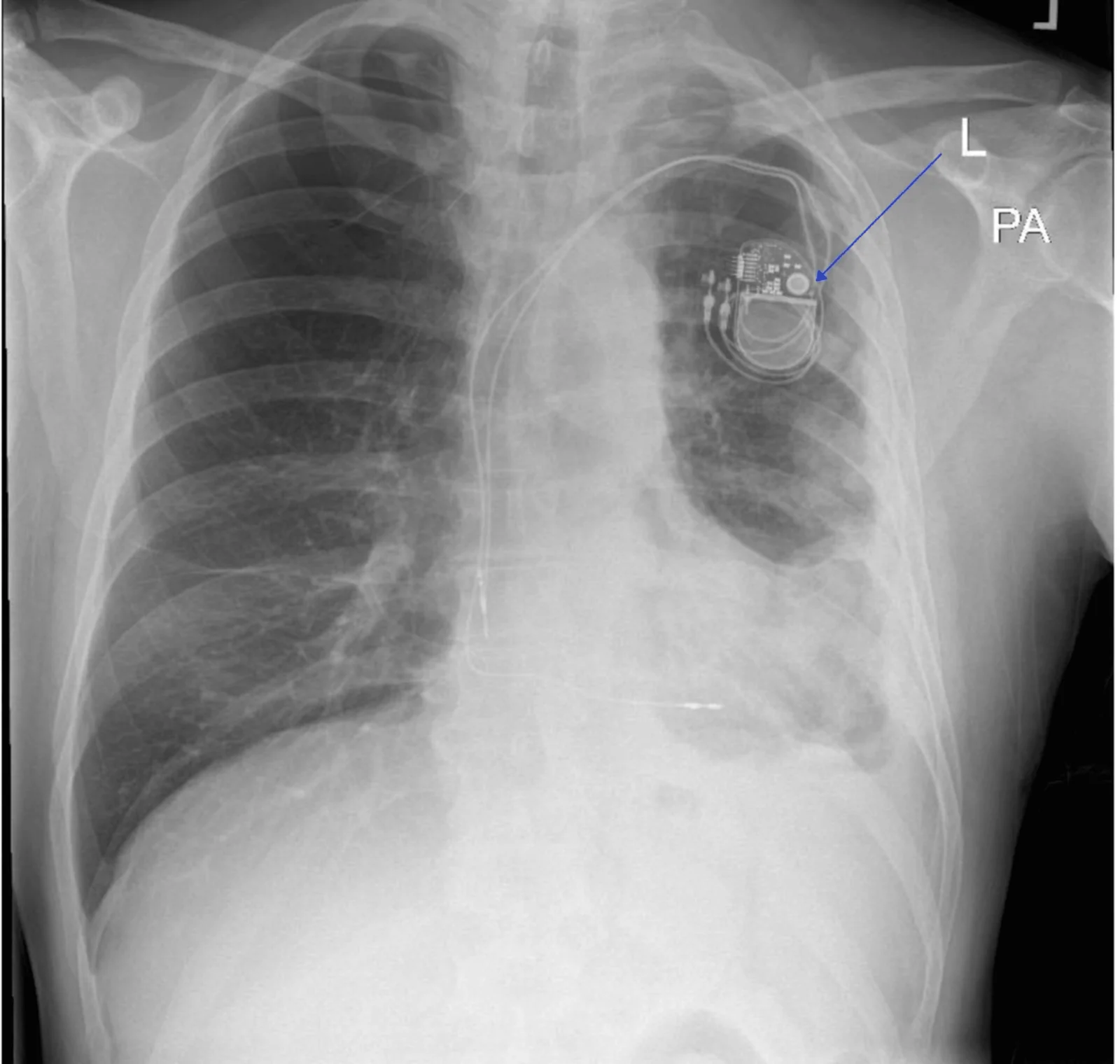
Chest X-ray showing a well-positioned dual chamber pacemaker in the left pectoral region (blue arrow)

The patient was transferred to a tertiary cardiac centre for urgent pacemaker insertion. A permanent dual-chamber pacemaker was successfully inserted (Figure 5), and the patient's heart rhythm stabilised. The GCS normalised by day five of admission. However, the patient had no recollection of events leading to or during initial hospital admission; he was also noticeably emotionally labile. The steroid weaning plan was discussed and agreed upon. Methylprednisolone was to be dropped first to 500 mg/day, then to 250 mg/day, followed by 150 mg/day, after which a switch would be made to oral prednisolone if the patient remained stable.

The patient's ALT started to rise by day seven of admission. The initial ALT was 75. However, this continued to rise, peaking at 258 on day 14 of admission. He then unexpectedly developed severe acute kidney injury (AKI) by day 17 of admission (Table 3). This coincided with IV methylprednisolone weaning down to 250 mg/day from 1000 mg/day. Urinalysis was positive for blood and protein; ANCA remained elevated, although titres for anti-myeloperoxidase and anti-proteinase-3 were negative. This was an unexpected setback because the patient was getting better, inflammatory markers (CRP and WCC) were normal; he was managing to eat and drink, and he was looking forward to being discharged home.

**Table 3:** Follow-up lab results on day 17 of hospital admission ALP: Alkaline phosphatase, ALT: Alanine transaminase, LDH: Lactate dehydrogenase, CK: Creatine kinase, ANA: Anti-nuclear antibody, ANCA: Anti-neutrophil cytoplasmic antibody, SG: Specific gravity, ACR: Albumin creatinine ratio

Test		Result	Reference range
Metabolic profile	Sodium	129	133-146 mmol/L
	Potassium	5.9	3.5-5.3 mmol/L
	Urea	20.2	2.5-7.8 mmol/L
	Creatinine	385	58-110 umol/L
	Albumin	36	35-55 g/L
	ALP	142	30-130 U/L
	ALT	130	<50 U/L
	Bilirubin	4	<21umol/L
	Lactate dehydrogenase (LDH)	713	<250 U/L
	Lactate	1.4	0.5-1.6 mmol/L
	Calcium	2.33	2.20-2.60 mmol/L
	Troponin T	48	<14 ng/L
Autoimmune screen	ANA	Negative	
	ANCA	1:160 (P-ANCA)	
	C4	0.27	0.14-0.54
	C3	1.66	0.75-1.65
Urine chemistry	Protein	+	
	Blood	+	
	Specific gravity (SG)	1.010	
	Albumin creatinine ratio (ACR)	8.7	<3 mg/mmol

**Figure 6:**
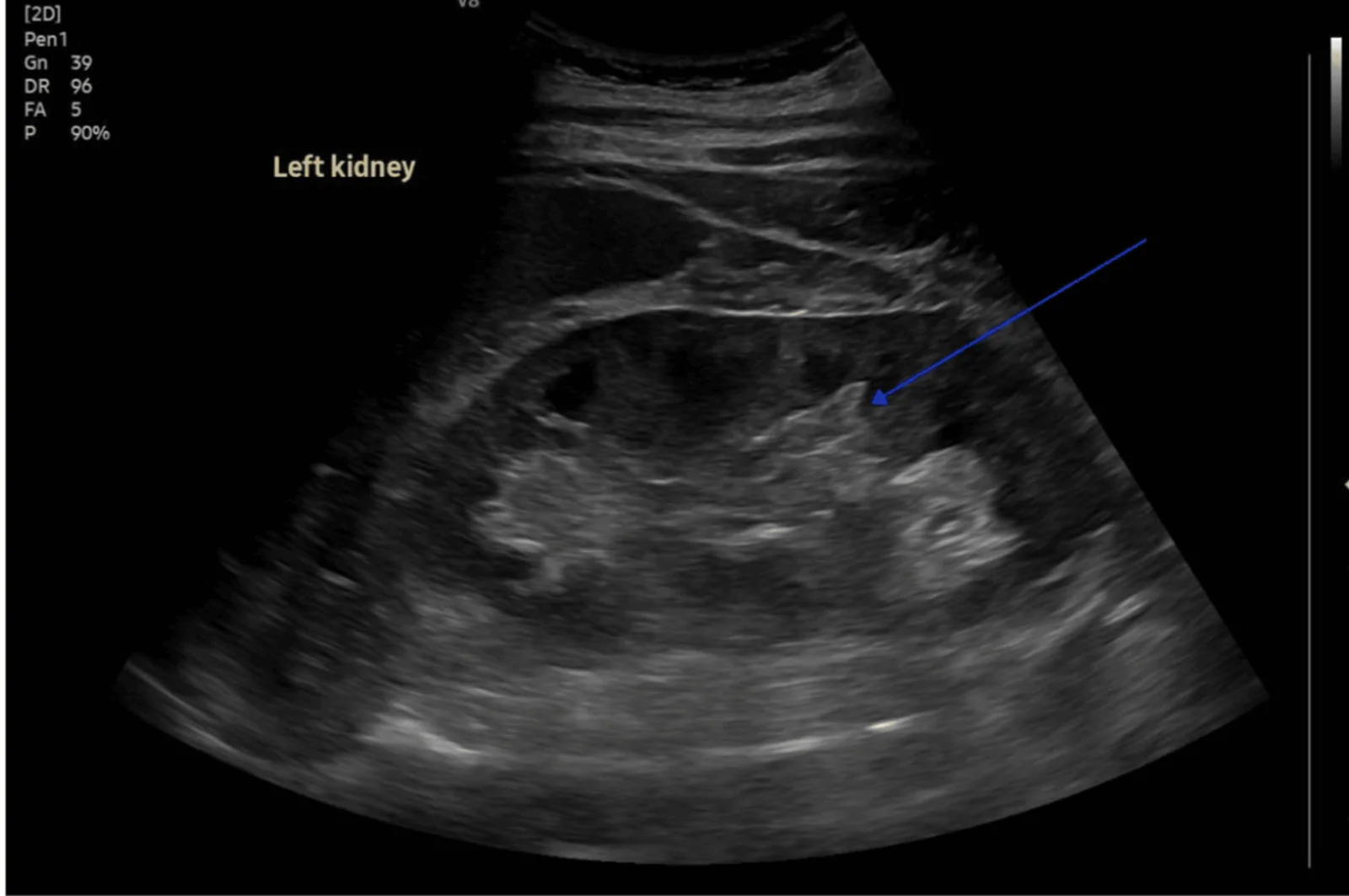
Ultrasound scan showing unobstructed left kidney with normal corticomedullary differentiation (blue arrow)

**Figure 7:**
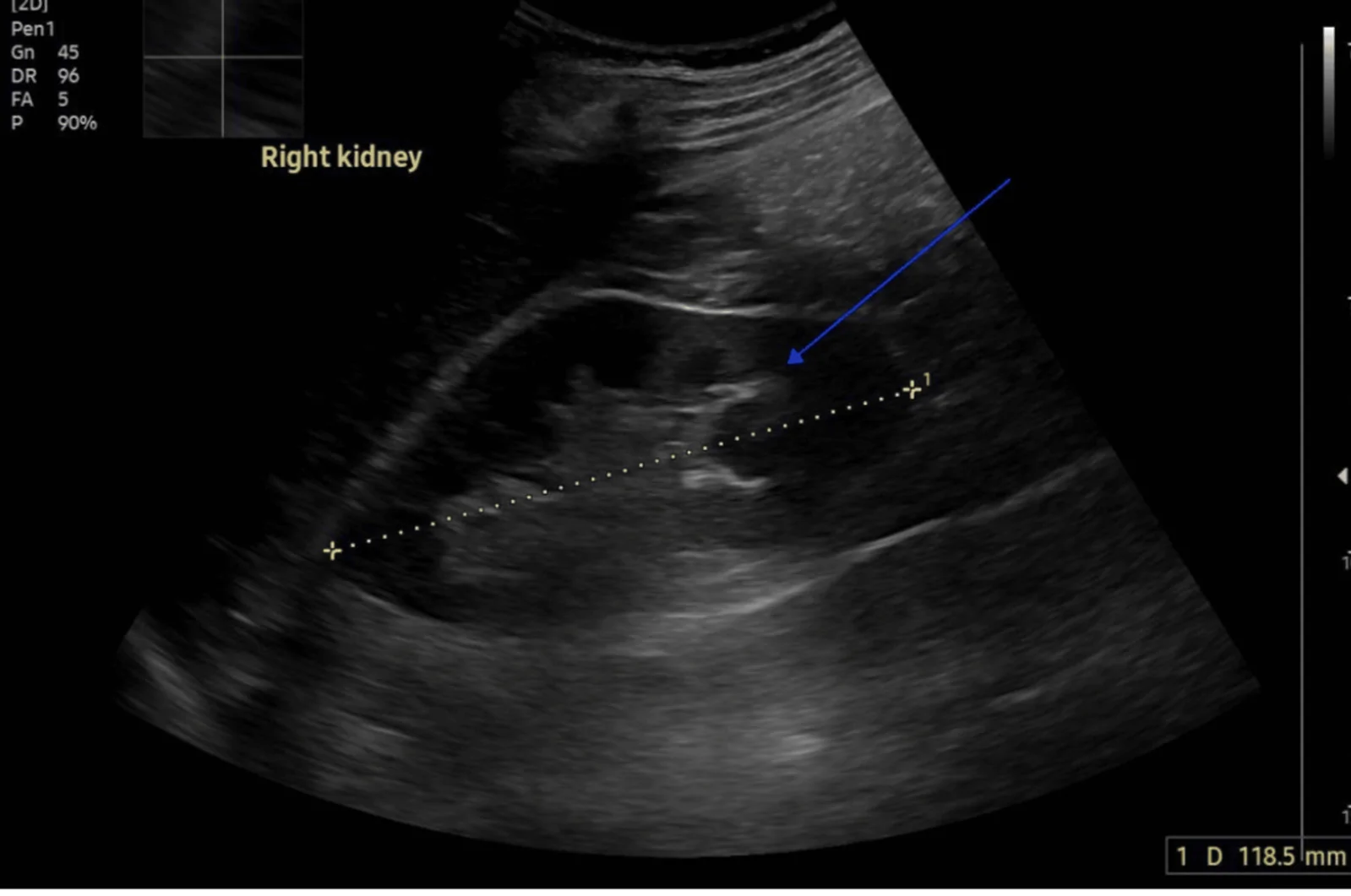
Ultrasound scan showing unobstructed right kidney with normal corticomedullary differentiation (blue arrow)

A detailed review was conducted for a possible cause of the patient's AKI. Ultrasound imaging of the urinary tract revealed no evidence of obstructive uropathy (Figures 6-7). Although CK was elevated initially during the course of the admission, it had normalised several days prior to abnormal kidney function; inflammatory markers and lactate were also normal. The timing of the AKI also overlapped with an increase in the patient's ALT, suggesting a possible immune-driven process affecting both the kidneys and liver. The increase in troponin was believed to be due to the severe AKI.

Lisinopril, which the patient had been on for years for treatment of hypertension, was stopped. Although the urea/creatinine ratio was 12.99, IV fluids were started. Methylprednisolone was re-escalated to 1000 mg/day, and the patient was booked for discussion in the immuno-oncology multidisciplinary team (IO-MDT) meeting. Kidney function improved rapidly with high-dose steroids, IV fluids and supportive treatment. The IO-MDT concluded that the patient's presentation, although atypical, was likely due to the unmasking of a pre-existing autoimmune tendency by ICIs and advised tapering steroids rapidly with a view to re-escalation if the patient relapsed. The patient remained stable throughout the rest of the admission and was subsequently discharged by day 24. The treating oncologist felt the cardiac event likely represented a grade 3+ irAE and discontinued atezolizumab permanently. The treating clinical team felt the encephalitis, AKI, and hepatitis all met the threshold for grade 3+ irAEs.

Although there was a plan to switch the patient to a tyrosine kinase inhibitor (TKI), this did not materialise as he developed a large pancreatic metastasis and functionally declined. The patient was deemed unsuitable for further systemic anticancer treatment (SACT) and was offered palliative radiotherapy to both the primary tumour site and pancreatic deposit for pain management. He eventually passed away six months after hospital discharge.

## Discussion

This complex case of sequential multiorgan immune activation following PD-L1 blockade highlights the shifting landscape of acute medicine in the era of immunotherapy. Immune checkpoint inhibitor toxicities are no longer niche; frontline physicians who are often the first to encounter these patients in the emergency setting need to have a comprehensive understanding of their mechanisms and spectrum to safely manage them [6]. Vigilance, comfort with navigating clinical uncertainty, and readiness to coordinate across multiple specialties are essential for good outcomes. 

This patient's presentation was atypical because the irAEs did not fit the pattern of well-established ICI toxicity overlap phenotypes, both in terms of the combination of organs affected and the sequence of their involvement. It is well established that ICI toxicity may affect virtually any organ system [6]. However, presentations that do not fit established toxicity phenotypes make diagnosis challenging by increasing the potential for diagnostic disputes across specialties. For example, in the case presented here, leptomeningeal disease was suspected, for which a lower dose of steroid was recommended. Interestingly, the clinical team who took over the patient's care by day three felt that a high-grade atrioventricular (AV) block with ventricular standstill captured on cardiac monitoring was sufficient to explain the patient's symptoms. Similarly, the nephrology team was hesitant to ascribe the subsequent AKI to an immune-mediated process. Overall, these suggest the threshold among clinicians for considering immunotherapy-related toxicity as a diagnosis remains relatively high. In our case, even when irAE was considered, there was significant hesitancy to consider multiorgan toxicity when the presentation didn't fit established phenotypes. Table 4 summarises the key timeline of events relative to atezolizumab infusion. 

**Table 4:** Timeline of clinical events during the patient's admission relative to the dose of atezolizumab NA: Not applicable, AKI: Acute kidney injury, CNS: Central nervous system

Clinical event	Organ system	Day of onset relative to atezolizumab dose	Steroid intervention context
First dose of atezolizumab infused	NA	Day 0	Not on steroids
Onset of seizures and encephalitis	CNS	Day 16	1 mg/kg/day of IV methylprednisolone commenced
Complete heart block/ventricular standstill discovered on cardiac monitoring	Heart	Day 19	IV methylprednisolone escalated to 1000mg/day
Transaminitis and AKI	Renal and hepatic	Day 32	Coincided with IV methylprednisolone tapering, rapidly improved with steroid re-escalation and supportive care

This patient’s clinical presentation is highly suggestive of ICI-associated irAEs for several reasons. First, the patient had multiple risk factors for ICI toxicity: male on PD-L1 therapy, age below 60, lung cancer (non-small cell lung cancer (NSCLC)), thoracic radiation prior to ICI commencement, history of asthma, autoantibodies (ANCA positive) and hypertension [7]. The patient’s history of asthma along with non-specific ANCA positivity is interesting because asthma can in some cases be the initial presentation of auto-inflammatory systemic conditions like eosinophilic granulomatosis with polyangitis (EGPA) [8]. While there was no clinical or histopathological evidence to support a diagnosis of EGPA in this case, the coexistence of asthma and ANCA positivity prior to the commencement of ICIs may suggest a predisposition toward immune dysregulation or a break in self-tolerance which could hypothetically be exacerbated by ICI exposure. Type 1 hypersensitivity reactions like asthma were also found to increase the risk of irAEs in solid tumours like NSCLC (hazard ratio: 1.48; 95% CI: 1.02-2.14; p = 0.037) [9]. We do acknowledge that direct evidence linking asthma to ICI-induced irAEs exists only for pneumonitis, and the non-specific nature of the ANCA subtype along with the absence of classic vasculitis features in this case suggests this assertion should be interpreted with caution and considered only as hypothesis-generating.

Second, the unfolding of this sequence of clinical events within 16 days of atezolizumab initiation, as highlighted in Table 4, establishes a clear temporal association with immune checkpoint blockade. Although the median time to onset of toxicity in patients receiving atezolizumab is 1.68 months, toxicities have been reported within days of infusion [10]. Finally, the absence of other well-defined autoimmune diseases or causes for multiorgan dysfunction despite extensive investigations makes ICI toxicity a plausible pathophysiologic explanation for the patient’s presentation. Although the markedly elevated lactate (10.4 mmol/L) and raised inflammatory markers at the time of initial presentation raised concerns for sepsis, this was never substantiated, as all microbial cultures were negative. The lactate elevation is plausibly explained by the patient’s generalised tonic-clonic seizures and status epilepticus, a well-recognised cause of transient hyperlactataemia. Importantly, by the time of subsequent development of renal and hepatic dysfunction, these parameters had normalised, arguing against ongoing sepsis as a unifying diagnosis. This temporal dissociation supports an evolving immune-mediated process rather than a single infective aetiology.

**Figure 8:**
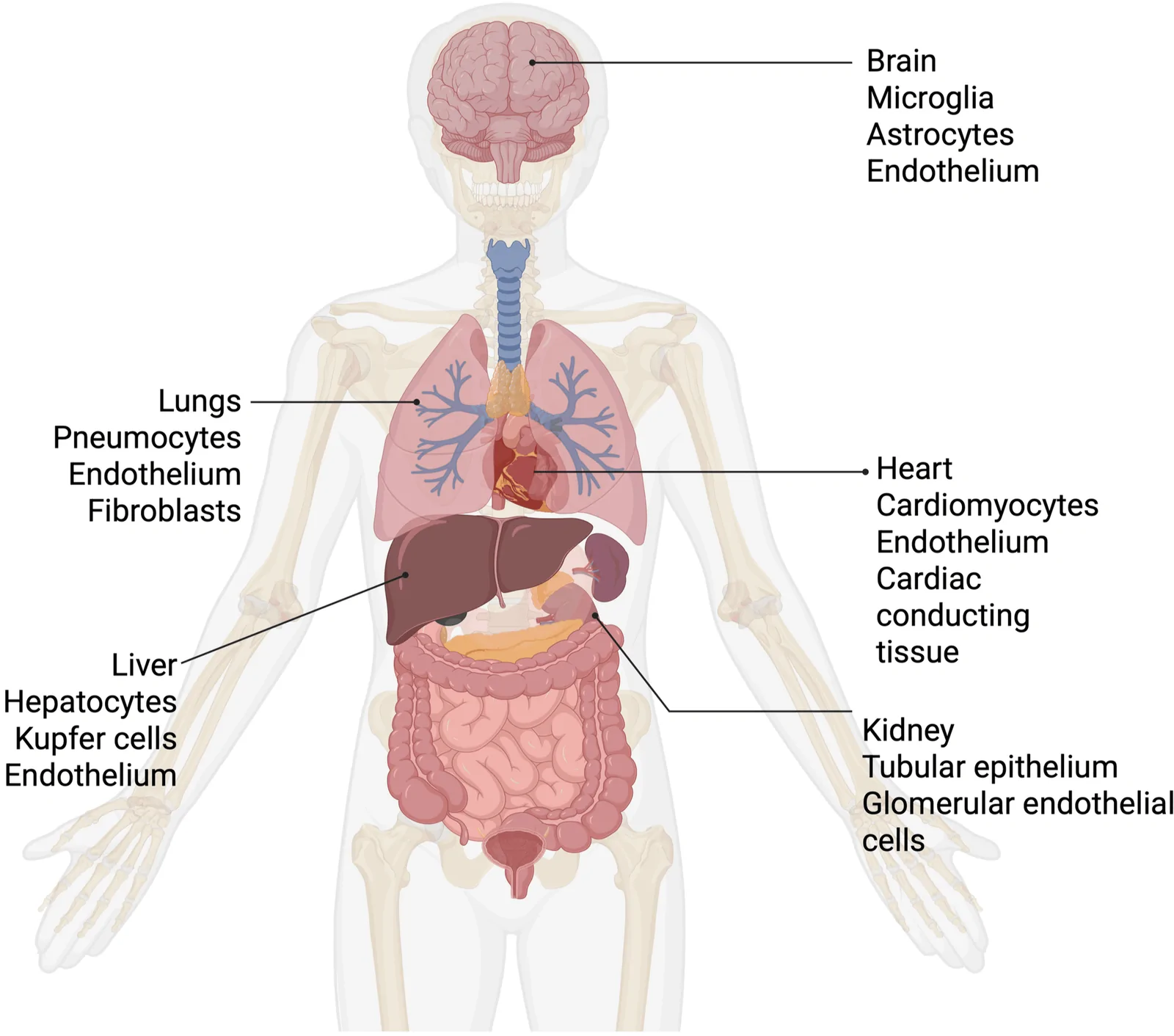
Illustration of major non-haematopoeitic tissues that constitutively and/or inducibly express PDL-1 and may contribute to tissue-specific immune tolerance Representative cell types expressing PDL-1 are shown for each organ. This original schematic was created by the authors using BioRender.com (BioRender; Toronto, ON, CAN) without the use of generative AI tools. PDL-1: Programmed death ligand 1

The organs involved (brain, heart, kidneys and liver) do share some metabolic and immunologic similarities. All four organs have rich vascular beds, enjoy a degree of immune privilege, and are metabolically very active with abundant mitochondria and high levels of oxidative phosphorylation, which predisposes them to oxidative stress. The organs each have an abundance of tissue-resident macrophages (TRMs) that can exist in both tolerogenic and pro-inflammatory phenotypes that can drive inflammation by releasing cytokines. Immune homeostasis in these organs is also heavily reliant on PD1/PDL-1 interactions. It is well established that PD-L1 is expressed by non-haematopoietic tissues (Figure 8), including renal tubular epithelium, hepatocytes, myocardial capillary endothelium, cardiac conduction tissue, and CNS glial cells, where it functions to locally restrain effector T cell response and maintain tissue-level tolerance [11-13].

The expression of PDL-1 is also upregulated in some tissues during periods of physiologic stress as a protective mechanism. By blocking these pathways, atezolizumab plausibly disinhibits autoreactive effector T-cells, promotes pro-inflammatory TRM phenotypes, and predisposes to tissue inflammation across these organs. Real-world data also suggests the pattern of toxicities observed in our patient may not be random. An analysis of irAEs data from one hospital system in the US published by the European Society for Medical Oncology (ESMO) found that encephalitis was associated with an increased risk of cardiac conduction disorders in patients on ICIs (HR, 4.35; 95% CI, 1.6-11.87) [9]. Nephritis was also associated with cardiovascular irAEs in the adjusted Cox proportional hazard model [14]. Sequential multiorgan toxicities resulting in death have also been previously reported in a retrospective study of delayed immunotherapy-related toxicities published by Owen et al., although a different combination of organs was involved [15]. 

Neurological immune-related adverse events (n-irAEs) are rare, severe side effects of immunotherapy affecting up to 1% to 5% of patients on checkpoint inhibitors. Encephalitis is the most frequent CNS toxicity induced by ICIs and represents 13% of all n-irAEs [11]. Approximately 50% of cases present as meningoencephalitis with altered mental status, memory deficits (especially short-term), psychiatric symptoms, fever, headache, and seizures [16]. Interestingly, encephalitis seems to occur more frequently in lung cancer patients receiving anti-PD-(L)1 therapy [17]. Although the median onset time is after three treatment cycles, very early and late presentations are common [17]. The clinical symptoms of encephalitis, however, are not specific to ICI-related encephalitis and may be seen with various other common causes of meningoencephalitis, such as viral and bacterial infections; this makes ruling out differential diagnoses an important initial step. In our case, this process was challenging, as the patient was clinically too unstable and the initial attempt at LP had to be abandoned. Although an MRI of the brain was obtained, it did not show the typical picture of encephalitis or leptomeningeal disease. Antibody testing for autoimmune encephalitis came back negative. It's noteworthy that a normal MRI and negative antibodies do not exclude the diagnosis of ICI-related encephalitis and should not be relied on as such [17,18]. This is more so with focal limbic encephalitis, which best fits our patient's presentation. Negative MRI and autoantibodies are, however, good prognostic factors [18,19].

The cardiac rhythm abnormalities that developed during the course of the patient's hospital admission are also noteworthy. The initial rhythm was a persistent sinus tachycardia with rates above 150 beats per minute. This was initially believed to be due to volume depletion, stress, hypoxia, and possible infection. However, the rate did not respond to IV fluids, oxygen, or antimicrobials and morphed into tachy-brady episodes and eventually high-grade AV block with ventricular standstill 72 hours after admission. While myocarditis is the most common and well-established cardiac irAE, it is becoming more apparent that arrhythmias can occur as a complication of myocarditis, endocrinopathies (thyrotoxicosis), or as standalone toxicities by themselves. A single-centre cohort experience of 89 cases of cardiovascular irAEs published by Andres et al. reported ICI-related myocarditis was the most frequent event (n = 33, 37%), followed by tachyarrhythmias (n = 27, 30%). Bradyarrhythmias were recorded in three patients, with one having a third-degree AV block requiring pacemaker implantation [20]. The overall incidence of cardiac arrhythmias is substantially higher than that of myocarditis (1.5% for conduction disorders vs 0.05% for myocarditis) according to real-world US clinical data published in 2021 [14].

While arrhythmic complications have traditionally been thought to result from underlying myocardial inflammation, it is possible that PD-1/PD-L1 blockade itself may have a direct impact on the development of arrhythmias. Cardiomyocytes, capillaries, and conduction tissue express PD-L1, which serves as a protective checkpoint limiting immune-mediated injury. The expression of PD-L1 is also upregulated in other models of cardiac injury and may be part of cardiomyocyte survival pathways independent of the checkpoint function [21]. Additional research is needed to fully understand the pathophysiology of ICI-induced arrhythmias. It is also possible that our patient developed myocarditis despite the minimal rise in cardiac biomarkers both at presentation and during initial hospital admission, as arrhythmias are often a harbinger of yet-to-be-diagnosed myocarditis [21].

While cardiac troponin and CK-MB fractions are useful in diagnosing ICI myocarditis, normal levels should not be used to exclude the diagnosis [21]. Where the diagnosis remains uncertain, endomyocardial biopsy remains the definitive diagnostic test. This, however, may not be practical outside of high-resource academic centres. Cardiac magnetic resonance (CMR) is the preferred non-invasive modality for the diagnosis of ICI myocarditis; however, its use as the gold standard for the exclusion of myocarditis in patients with high clinical suspicion has been called into question due to low sensitivity. Late gadolinium enhancement and elevated T2-weighted short tau inversion recovery (STIR) signal, which make up the modified Lake-Louise criteria, were present in only 48% and 28% of patients, respectively, from an international registry of patients with ICI-associated myocarditis [21]. The CMR is also logistically challenging in resource-constrained settings. 

**Figure 9:**
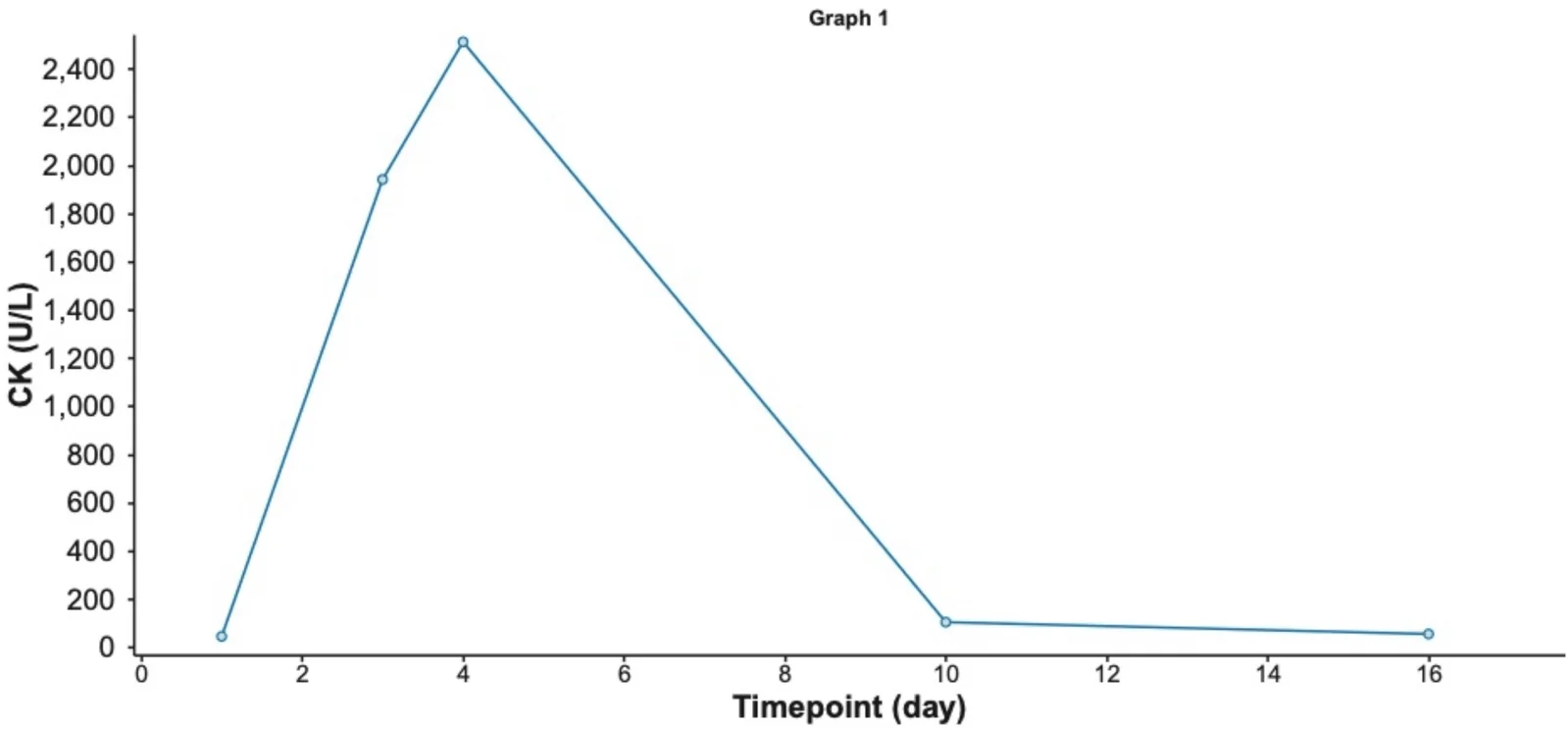
CK trend throughout admission showing normalisation prior to AKI onset AKI: Acute kidney injury, CK: Creatine kinase

**Figure 10:**
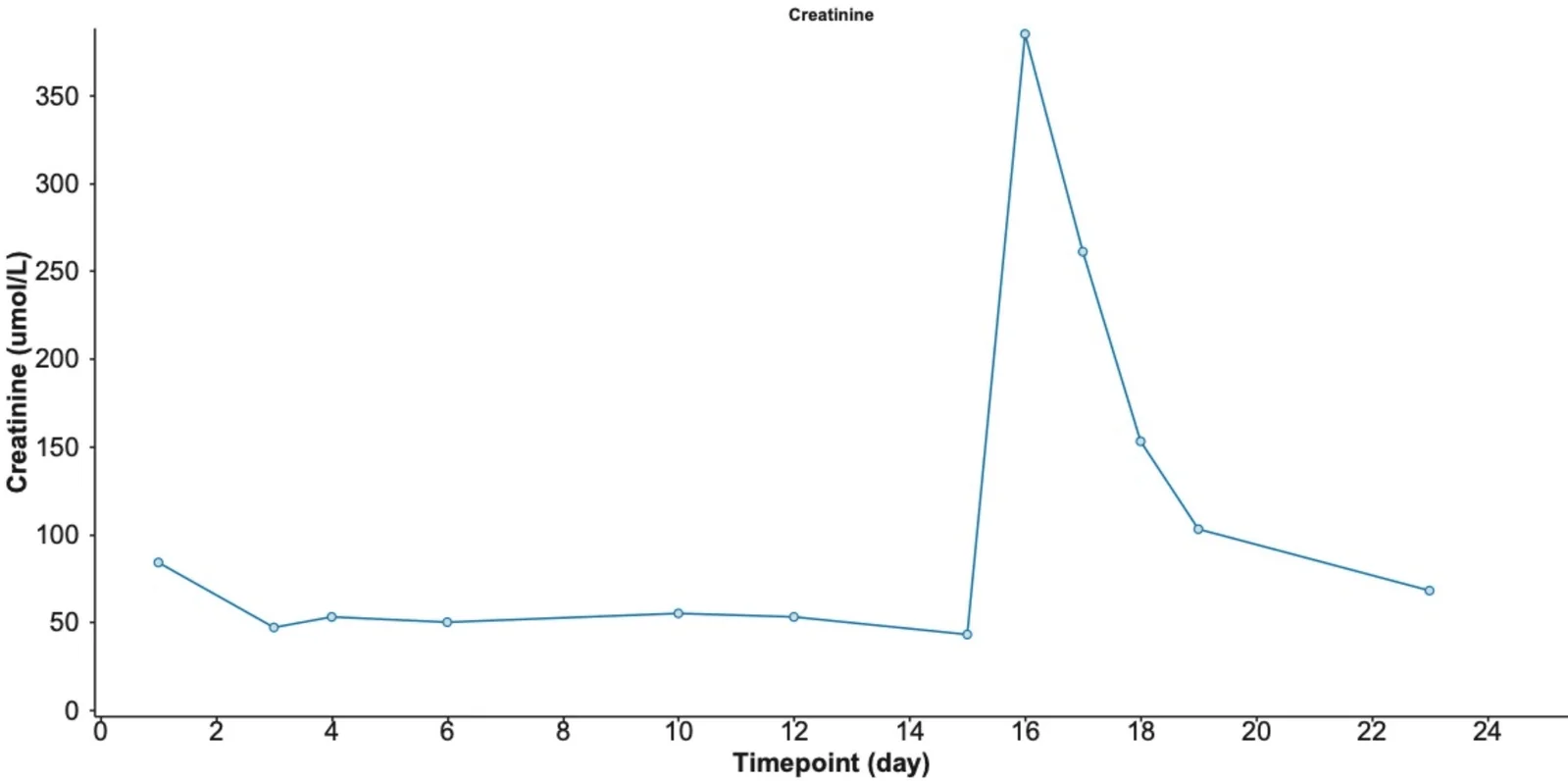
Creatinine trend throughout admission showing AKI onset and subsequent resolution AKI: Acute kidney injury

**Figure 11:**
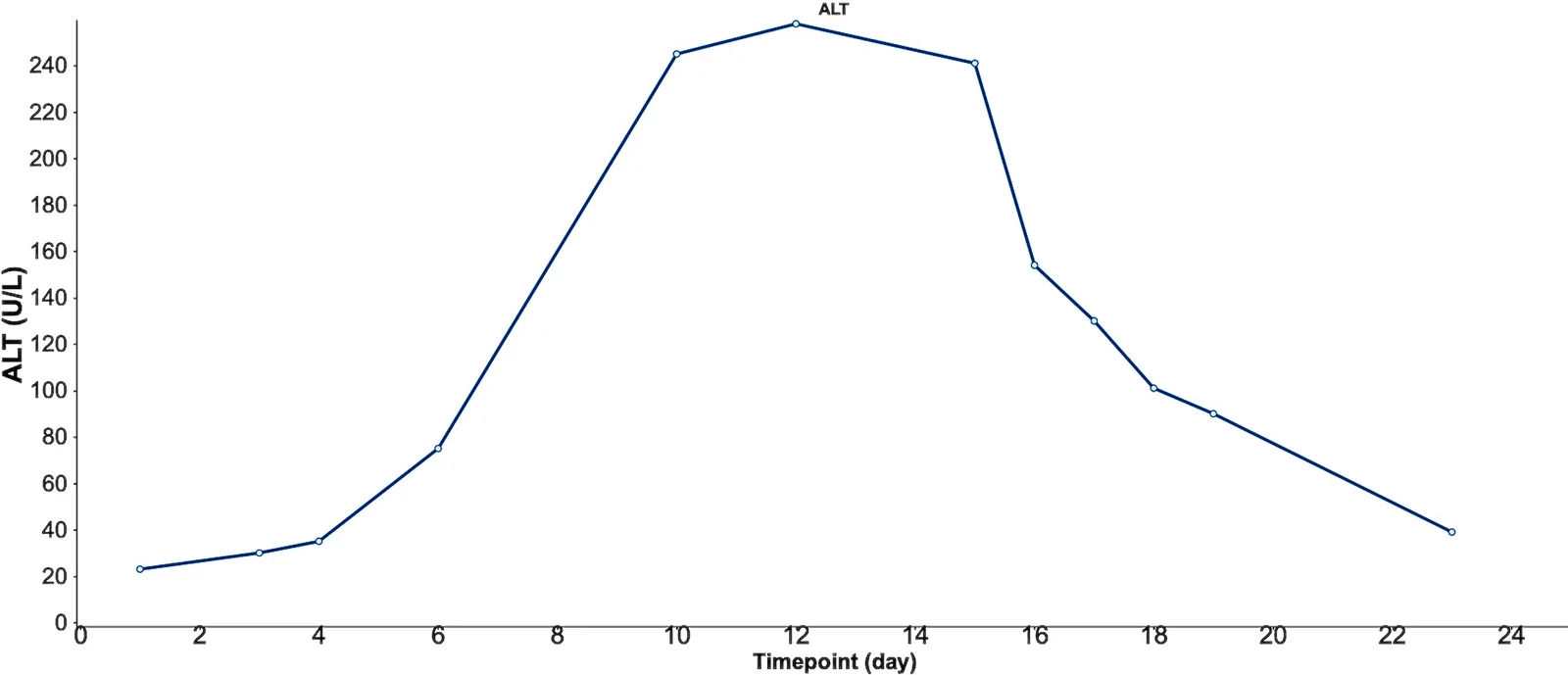
Full ALT trend throughout the time of admission showing transaminitis that worsened after steroid taper and resolved after steroid re-escalation ALT: Alanine transaminase

The patient's severe AKI 17 days post-admission was another phenomenon we could not easily explain. Urinalysis was positive for both protein and blood, suggesting possible intrinsic renal inflammation. The timing of AKI onset, which overlapped with the rise in liver enzymes, is noteworthy. It suggests a possible immune-driven inflammatory process affecting the two organs. Although the patient was on IV acyclovir, he was well hydrated and had been on it for one week; acyclovir-induced acute tubular necrosis is also more likely to occur within 72 hours of initiation. The patient's CK had fully normalised several days before AKI onset, ruling out rhabdomyolysis as a cause of his AKI. The line charts for CK in Figure 9 and creatinine in Figure 10 show a clear divergence in the trends of CK and creatinine during the time of admission. Renal impairment also coincided with a significant reduction in methylprednisolone dose as the patient was being weaned. Both liver and renal function immediately improved with high-dose methylprednisolone along with supportive measures, suggesting the underlying cause was probably immune-mediated. The line chart in Figure 11 shows the trend of ALT during admission.

Finally, to strengthen causal inference, we applied structured assessment tools. Using the Naranjo Adverse Drug Reaction Probability Scale [22], a score of 4-5 was obtained, indicating a 'possible' to 'probable' relationship between atezolizumab and the observed multiorgan toxicity. Similarly, application of the WHO-Uppsala Monitoring Centre (UMC) causality framework [23] classified the association as 'possible', reflecting a strong temporal relationship and steroid responsiveness but incomplete exclusion of alternative diagnoses such as infection or paraneoplastic processes. These findings support, but do not definitively establish, an ICI-mediated mechanism. However, it is noteworthy that while structured causality tools such as the Naranjo scale and WHO-UMC criteria [22, 23] were applied to enhance methodological rigour, their applicability to ICI-related adverse events is inherently limited. These frameworks were developed for conventional pharmacological toxicities characterised by dose dependence and predictable temporality, whereas irAEs represent immune-mediated phenomena with variable latency, multiorgan involvement, and responses driven by immunosuppression rather than simple drug withdrawal. Consequently, such tools may underestimate the likelihood of causality in this context. Greater diagnostic weight was therefore placed on clinical phenotype, temporal evolution, multiorgan involvement, and response to corticosteroid therapy, in line with current oncology practice.

This case report has several important limitations that warrant consideration. First, the absence of definitive diagnostic testing across multiple organs reduces the certainty of causal attribution. The LP was not performed due to clinical instability, limiting exclusion of infectious or alternative autoimmune causes of encephalitis. Electroencephalography (EEG) was not done due to the patient's acuity, progression of symptoms, and logistical challenges. Similarly, cardiac MRI and endomyocardial biopsy were not undertaken, precluding confirmation of myocarditis as the underlying substrate for the observed conduction abnormalities. Renal and hepatic involvement were inferred clinically, as tissue diagnosis was not obtained, and competing aetiologies, including drug-induced injury and sepsis-related organ dysfunction, although unlikely, cannot be fully ruled out. Second, although the temporal association with atezolizumab exposure and the observed steroid responsiveness, including relapse during tapering, strongly support an immune-mediated process, a causal relationship cannot be established. Third, the presence of non-specific autoimmune markers, such as ANCA positivity, raises the possibility of pre-existing immune dysregulation; however, the clinical significance of these findings remains uncertain. Finally, as a single case report, the generalisability of these observations is inherently limited, and caution must be exercised in extrapolating mechanistic or clinical conclusions. Nonetheless, this case highlights a plausible and clinically important pattern of sequential multiorgan toxicity following immune checkpoint inhibition, which warrants further investigation in larger cohorts.

## Conclusions

There is no doubt ICIs have dramatically improved outcomes across diverse cancer types in all treatment settings. The number of patients receiving these therapies globally will continue to increase as they continue to gain regulatory approval for use in an expanding list of cancers. The incidence of their related toxicities will no doubt also increase. It is expected that ICIs will continue to reshape the landscape of acute internal medicine. Frontline clinicians should be aware that sequential toxicity phenotypes may also occur and could pose significant diagnostic challenges. This requires ongoing vigilance and cross-speciality coordination.
